# The GABA_A_ antagonist bicuculline attenuates progesterone-induced memory impairments in middle-aged ovariectomized rats

**DOI:** 10.3389/fnagi.2015.00149

**Published:** 2015-08-14

**Authors:** B. Blair Braden, Melissa L. Kingston, Elizabeth N. Koenig, Courtney N. Lavery, Candy W. S. Tsang, Heather A. Bimonte-Nelson

**Affiliations:** ^1^Human Brain Mapping Laboratory, Neuroimaging, Barrow Neurological Institute, St. Joseph’s Hospital and Medical CenterPhoenix, AZ, USA; ^2^Memory and Aging Laboratory, Department of Psychology, Arizona State UniversityTempe, AZ, USA; ^3^Arizona Alzheimer’s ConsortiumPhoenix, AZ, USA

**Keywords:** progesterone, memory, aging, GABA, bicuculline, hormone therapy, menopause

## Abstract

In women, high levels of natural progesterone have been associated with detrimental cognitive effects via the “maternal amnesia” phenomenon as well as in controlled experiments. In aged ovariectomized (Ovx) rats, progesterone has been shown to impair cognition and impact the GABAergic system in cognitive brain regions. Here, we tested whether the GABAergic system is a mechanism of progesterone’s detrimental cognitive effects in the Ovx rat by attempting to reverse progesterone-induced impairments via concomitant treatment with the GABA_A_ antagonist, bicuculline. Thirteen month old rats received Ovx plus daily vehicle, progesterone, bicuculline, or progesterone+bicuculline injections beginning 2 weeks prior to testing. The water radial-arm maze was used to evaluate spatial working and reference memory. During learning, rats administered progesterone made more working memory errors than those administered vehicle, and this impairment was reversed by the addition of bicuculline. The progesterone impairment was transient and all animals performed similarly by the end of regular testing. On the last day of testing, a 6 hour delay was administered to evaluate memory retention. Progesterone-treated rats were the only group to increase working memory errors with the delay relative to baseline performance; again, the addition of bicuculline prevented the progesterone-induced impairment. The vehicle, bicuculline, and progesterone+bicuculline groups were not impaired by the delay. The current rodent findings corroborate prior research reporting progesterone-induced detriments on cognition in women and in the aging Ovx rat. Moreover, the data suggest that the progesterone-induced cognitive impairment is, in part, related to the GABAergic system. Given that progesterone is included in numerous clinically-prescribed hormone therapies and contraceptives (e.g., micronized), and as synthetic analogs, further research is warranted to better understand the parameters and mechanism(s) of progesterone-induced cognitive impairments.

## Introduction

Within their lifetime, women will have to make the decision of whether to take exogenous hormones, either for contraception or for hormone therapy (HT) associated with menopause. Women that have a uterus must include a progestogen in their HT regimen to protect against endometrial hyperplasia (Smith et al., 1975). The most commonly prescribed progestin component of HT, and the sole hormone component of the contraceptive Depo Provera, medroxyprogesterone acetate (MPA), has been associated with memory impairments in rodent models across the adult lifespan (Braden et al., 2010, 2011; Lowry et al., 2010; Okojie and Oyekunle, 2014) and adverse *in vitro* cellular effects (Nilsen and Brinton, 2002b; Nilsen et al., 2006). There is evidence in postmenopausal women that combination HT treatment including MPA increases the risk of dementia (Shumaker et al., 2003; Coker et al., 2010). In women, like MPA, natural progesterone is associated with detrimental effects on cognition in both the phenomenon of “maternal amnesia” (Brett and Baxendale, 2001) and in controlled studies of healthy women receiving oral progesterone (Freeman et al., 1992; van Wingen et al., 2007). Progesterone can impair memory in young and aged rodent models (Bimonte-Nelson et al., 2004b; Braden et al., 2010; Sun et al., 2010) and reverse the beneficial effects of 17β-estradiol *in vivo* (Bimonte-Nelson et al., 2006; Harburger et al., 2007; but see Gibbs, 2000; Markham et al., 2002) and *in vitro* (Nilsen and Brinton, 2002a; Aguirre and Baudry, 2009). Further, administration of allopregnanolone, a progesterone metabolite, has been shown to impair cognition in healthy women (Kask et al., 2008) and young rats (Frye and Sturgis, 1995; Ladurelle et al., 2000; Johansson et al., 2002; Rabinowitz et al., 2014). These findings are particularly salient since the use of progesterone (specifically, natural progesterone in its micronized form to be orally bioavailable) as the progestogen component of HT has become an option for women in the United States as recently as 1999 (Langer, 1999) and is a promising new candidate to replace MPA (Sturdee et al., 2011; The Writing Group for the PEPI Trial, 1995; Langer, 1999).

Preclinical studies indicate that a potential mechanism for progesterone-induced memory impairments includes the GABAergic system. Several metabolites of progesterone have a high affinity for the GABA_A_ receptor wherein they can act as positive allosteric modulators (Paul and Purdy, 1992). Further, administration of progesterone decreases glutamic acid decarboxylase (GAD, the synthesizing enzyme and the rate limiting step of GABA production), protein levels (Braden et al., 2010) and mRNA activity (Wallis and Luttge, 1980), and can alter mRNA expression of subunits of the GABA_A_ receptor, in the hippocampus of ovariectomized (Ovx) rats (Weiland and Orchinik, 1995; Pazol et al., 2009). Further, in young Ovx mice, treatment with a GABA_A_ receptor antagonist attenuates the impairing effect of progesterone on an emotional learning task (Farr et al., 1995). It remains to be determined, however, whether the GABAergic system is a mechanism of progesterone’s effects on cognition during aging in the Ovx rat. We hypothesize that progesterone administration impairs cognition in the Ovx middle-aged or aged rat through an increase in GABA-mediated inhibition in the hippocampus, likely via its metabolites (Lan and Gee, 1994). Subsequently, an increase in GABA-mediated inhibition could impair memory by inhibiting the induction phase of long-term potentiation (LTP; Izquierdo et al., 1993). We propose to address the question of whether progesterone’s effects on memory are, in part, due to GABA_A_ modulation by administering the GABA_A_ receptor antagonist, bicuculline, to progesterone-treated middle-aged surgically menopausal animals. We predict that bicuculline will prevent progesterone’s negative effects on cognition. Investigating the GABAergic system’s relationship to progesterone’s cognitive effects in an animal model is a critical step in answering questions regarding the putative mechanisms by which natural progesterone impairs cognition. With this knowledge, we can move forward in the field of behavioral endocrinology studying progestogens that do not share these pharmacological characteristics, and are thus potentially safer alternatives to be used clinically for brain health and function.

## Materials and Methods

### Subjects

The subjects used in this study were 37, 13-month old Fischer-344 virgin female rats. Animals were born and reared within the aging colony at the National Institute on Aging at Harlan Laboratories (Indianapolis, IN, USA). Rats had access to food and water ad-lib, were maintained on a 12 h light/dark cycle, and were acclimated for several weeks before surgery was initiated at the Arizona State University animal facility. All procedures used in this study were approved by the local IACUC committee, and they adhered to NIH standards.

### Hormone Treatment and Ovariectomy

Rats were randomly assigned to one of four treatment groups: Ovx+Vehicle, Ovx+Progesterone (Ovx+Prog), Ovx+Bicuculline (Ovx+Bic), or Ovx+Prog+Bic. At the age of 13 months, all rats received isofluorane inhalation anesthesia, underwent dorsolateral incisions made bilaterally in the skin and peritoneum, and the ovaries and the tips of uterine horns were ligatured and removed. Next, muscle and skin were sutured. Beginning two days after surgery all drugs were administered via two daily subcutaneous injections in the scruff of the neck. For the first injection given daily, animals received either a control vehicle solution [0.4 mL sesame oil + 0.02 mL dimethyl sulfoxide (DMSO), both chemicals were from Sigma-Aldrich, St. Louis, MO, USA] or progesterone (0.7 mg, Sigma-Aldrich St. Louis, MO, USA; dissolved in 0.4 mL sesame oil + 0.02 mL DMSO). The progesterone dose was chosen as the daily equivalent of what animals received via osmotic subcutaneous pumps in Braden et al. (2010) whereby progesterone impaired memory in aged Ovx rats. For the second injection given daily, animals received either a control vehicle solution (sesame oil + 10% DMSO) or bicuculline (3.5 mg/kg; dissolved in sesame oil + 10% DMSO). Importantly, we determined that this dose of bicuculline was subthreshold for clinically visible signs of seizures in a different cohort of animals prior to initiation of the current study, corresponding with findings shown by other investigators (Brioni and McGaugh, 1988). Injections were continued through all behavior testing (approx. 30 m prior to testing) until sacrifice occurred.

### Vaginal Smears and Uterine Weights

Vaginal smears were evaluated 12 and 13 days after Ovx (and therefore after hormone and/or drug administration began). They were classified as proestrus, estrous, metestrus or diestrous based on prior protocols (Goldman et al., 2007; Acosta et al., 2009). At the time of sacrifice, the uterus of each subject was extracted, visible fat was trimmed, and the remaining tissue was immediately weighed (wet weight), in order to assess the impact of the hormone and drug on uterine tissues (Braden et al., 2010, 2011).

### Water Radial-Arm Maze

Thirteen days after initiation of treatment subjects began maze testing, with timing similar to previous studies in our laboratory (Talboom et al., 2010). Subjects were tested on the water radial-arm maze (WRAM) for 15 days to test spatial working and reference memory, including performance as working memory load increases, as described in prior publications (Bimonte and Denenberg, 2000; Bimonte et al., 2000, 2002). The maze had escape platforms hidden under the surface of the water at the ends of four of the eight radiating arms. A subject was assigned different platform locations which remained fixed for the duration of testing. Each rat was released from the start arm and had 3 m to find a hidden platform. Once a platform was located, the animal remained on it for 15 s, and was returned to its warmed cage for a 30 s inter-trial interval (ITI) until its next trial began. The just-chosen platform was then removed from the maze during the ITI. Next, the subject was again placed into the start alley and was allowed to locate another platform. For each rat, a session within a day was composed of four trials, with the number of platformed arms reduced by one for each subsequent trial. Thus, working memory was increasingly taxed as the trials progressed within a day, allowing us to test the ability to handle an increasing working memory load. Each subject was given one session a day for 14 consecutive days. On Day 15, a 6 h delay was administered between Trials 2 and 3, as done previously in our laboratory for middle-age animals on other water-escape tasks (Engler-Chiurazzi et al., 2011, 2012).

Quantification and blocking of errors were based on prior studies (Hyde et al., 1998, 2000; Bimonte and Denenberg, 2000; Bimonte et al., 2000, 2002). When the tip of a rat’s snout reached a mark demarcated on the outside of the arm (11 cm into the arm), an arm entry, or error, was counted. Errors were computed as done previously in WRAM studies (Bimonte et al., 2000, 2002; Hyde et al., 2000), and were quantified into working and reference memory errors (Jarrard et al., 1984). Working Memory Correct (WMC) errors were defined as the number of first and repeat entries into an arm from which a platform had been removed during that daily session. For WMC errors, Trial 1 was not included in analyses because a platform had not yet been removed. Reference Memory (RM) errors were defined as the number of first entries into an arm that never had a platform, and Working Memory Incorrect (WMI) errors were repeat entries into a RM arm. Our traditional protocol for evaluating treatment effects on the WRAM includes testing for 11–12 days and blocking errors into two blocks of 5–6 days (Bimonte and Denenberg, 1999; Bimonte et al., 2000; Braden et al., 2011). In the current study, however, animals required 14 days of regular testing to reach asymptotic performance, most likely due to the increased stress of multiple subcutaneous injections 30 m before test. Thus, to most thoroughly evaluate performance as learning progressed, errors were combined into three blocks (Block 1 = days 1–4, Block 2 = days 5–9, Block 3 = days 10–14).

### Visible Platform Maze

To confirm that all rats could execute the procedural task components of a water-escape task without difficulty, every animal was tested on a visible platform water-escape maze, as done previously (Braden et al., 2010, 2011). A rectangular tub (39 × 23 in) was filled with clear water and had a visible black platform (10 cm wide) elevated above the surface of the water. Extramaze cues on the walls were covered with opaque curtains. Rats were dropped off at the same maze location across trials, and the location of the platform for each trial varied in space semi-randomly (e.g., the platform was moved across trials). Subjects were required to locate the protruding platform, and were given 6 trials in 1 day. Maze performance was evaluated by latency (s) to the platform. Animals were sacrificed 3 days after visible platform maze testing.

### Statistical Analyses

Data were analyzed separately for each maze. To evaluate learning and potentially complex higher order Treatment group interactions with Days and/or Trials, an omnibus repeated measures ANOVA including all groups, with Treatment as the between variable, and blocks of Days and/or Trials as the within variable/s, was used. For uterine weights, Treatment effects were assessed via ANOVA. When ANOVA yielded a significant effect of Treatment, two group comparisons using *t*-tests were used to investigate group differences. For all statistical analyses, alpha was set as two-tailed and at 0.05, except for when assessing WRAM working memory (WMC and WMI) error differences on trial 4 alone, the trial with the highest working memory load, between Ovx+Prog and Ovx+Vehicle. For these assessments the test was one-tailed, as they were a replication from Bimonte-Nelson et al. (2004b) and Braden et al. (2010).

## Results

### Water Radial-Arm Maze

For the omnibus ANOVA, across all testing days (Days 1–14) and including all treatment groups, there was a main effect of Day for each error type (WMC: *F*_(13,429)_ = 5.828; *p* < 0.0001; WMI: *F*_(13,429)_ = 11.638; *p* < 0.0001; RM: *F*_(13,429)_ = 12.839; *p* < 0.0001), with errors decreasing across days, demonstrating learning.

**Figure 1:**
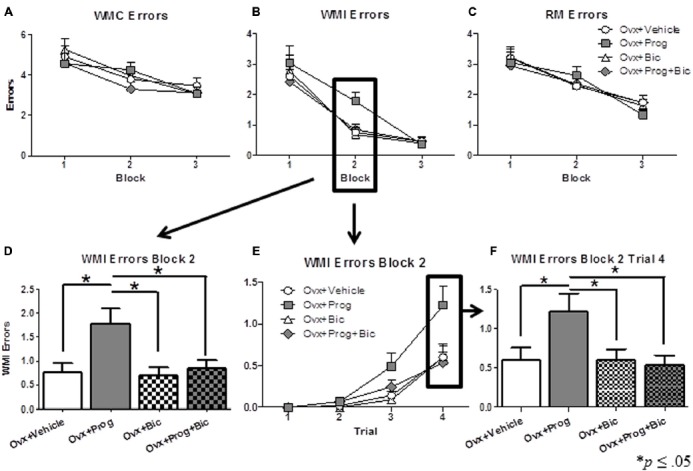
**Mean error scores (+SE) on the water-radial arm maze. (A)** Working Memory Correct (WMC) error scores, **(B)** Working Memory Incorrect (WMI) error scores, and **(C)** Reference Memory (RM) error scores across all days of regular testing in 4–5 day blocks. **(D)** WMI error scores on Block 2. **(E)** WMI error scores on Trials 1–4 on Block 2. **(F)** WMI error scores on Trial 4 on Block 2. *p < 0.05.

Figures 1A–C show mean errors scores (+SE) for each block for WMC, WMI, and RM, respectively, for each treatment group. For WMI, there was a significant omnibus ANOVA main effect of Treatment for Block 2 [*F*_(3,33)_ = 5.221; *p* < 0.005 (Figure 1D)]. Progesterone impaired performance as evidenced by the Ovx+Prog group committing more errors than the Ovx+Vehicle [*t*_(17)_ = 2.727; *p* < 0.05] and Ovx+Bic [*t*_(16)_ = 3.093; *p* < 0.01] groups. The addition of bicuculline reversed progesterone-induced working memory impairment as evidenced by the Ovx+Prog+Bic group committing fewer errors than the Ovx+Prog group [*t*_(16)_ = 2.435; *p* < 0.05] and performing similarly to the Ovx+Vehicle group. Within this block, there was also a significant Trial x Treatment interaction for WMI errors [*F*_(9,99)_ = 2.621; *p* < 0.01 (Figure 1E)]. At the highest working memory load (Trial 4) in Block 2, there was a main effect of Treatment [*F*_(3,33)_ = 3.393; *p* < 0.05 (Figure 1F)], with the Ovx+Prog group committing more WMI errors than all other treatment groups [Ovx+Prog vs. Ovx+Vehicle: *t*_(17)_ = 2.053; *p* < 0.05; Ovx+Prog vs. Ovx+Prog+Bic: *t*_(16)_ = 2.349; *p* < 0.05; Ovx+Prog vs. Ovx+Bic: *t*_(16)_ = 2.094; *p* = 0.05 (Figure 1F)]. These working memory load effects support a progesterone-induced working memory impairment that is reversed by the addition of bicuculline. There were no significant Treatment effects or interactions for WMI errors during Blocks 1 or 3, and no significant Treatment effects or interactions for WMC or RM errors during any block.

**Figure 2:**
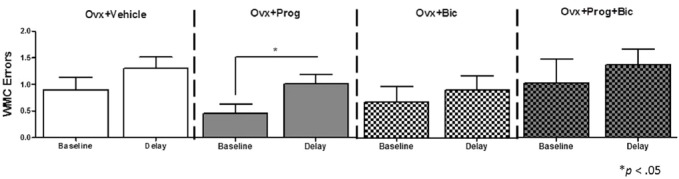
**Mean WMC error scores (+SE) on the water radial-arm maze for baseline (last day of regular testing Trial 3) vs. delay (Trial 3 immediately following a 6 h delay).** *p < 0.05.

After regular testing, a 6 h delay was administered between Trials 2 and 3. For WMC errors, we compared baseline performance on Trial 3 on Day 14 (the last day of regular testing) to Trial 3 performance immediately after the delay on Day 15, within each group. There was no statistically significant difference between groups on Trial 3 Day 14 baseline performance [*F*_(3,33)_ = 0.685; *p* = 0.568 (Figure 2)]. The Ovx+Prog group was the only group to significantly increase errors after the delay [Ovx+Prog, baseline vs. delay: *t*_(8)_ = 3.162; *p* < 0.05], demonstrating a susceptibility to delay-induced working memory impairments in progesterone-treated animals (Figure 2). The progesterone-induced impairment was obviated with the addition of bicuculline, as the Ovx+Prog+Bic group did not show a delay-induced impairment [Ovx+Prog+Bic, baseline vs. delay: *t*_(8)_ = 0.634; *p* = 0.54]. No group displayed a delay-induced increase in WMI or RM errors.

### Visible Platform Maze

**Figure 3:**
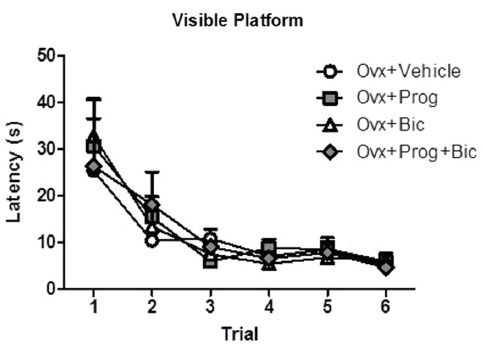
**Mean latency scores in seconds (+SE) on the visible platform maze**.

For the visible platform task, there was a main effect of Trial [*F*_(5,165)_ = 16.642; *p* < 0.0001 (Figure 3)], with Latency decreasing across all trials, demonstrating task learning. The omnibus ANOVA Treatment effect was not significant for Latency to the visible platform. By the fourth trial, all treatment groups found the visible platform within 9 s, confirming visual and motor competence to perform a water-escape swim task.

### Vaginal Smears

Twelve and 13 days after Ovx, vaginal smears were taken and classified as proestrus, estrous, metestrus or diestrous phases, per prior protocols (Goldman et al., 2007; Acosta et al., 2009). As expected, regardless of treatment, all rats showed leukocytic smears, indicative of a lack of vaginal stimulation (Braden et al., 2010).

### Uterine Weights

The omnibus ANOVA Treatment effect was not significant for uterine weight indicating that neither the progesterone nor bicuculline impacted this variable, replicating a lack of progesterone effect on uterus shown previously (Braden et al., 2010; Table 1).

**Table 1:** **Mean uterine weights in g (±SE)**.

	Ovx+Vehicle	Ovx+Prog	Ovx+Bic	Ovx+Prog+Bic
Uterine Weight (g)	0.169 ± 0.04	0.205 ± 0.02	0.194 ± 0.03	0.189 ± 0.01

## Discussion

Natural progesterone (in its micronized form) is a clinically used component of both contraceptives and HT, and has been discussed to be a promising new candidate to replace the synthetic progestin, MPA, as the progestogen component in HT (Sturdee et al., 2011; The Writing Group for the PEPI Trial, 1995; Langer, 1999). The results herein add to a growing body of literature implicating both MPA and natural progesterone as detrimental to cognition in the middle-aged to aged surgically menopausal rat (Bimonte-Nelson et al., 2004b, 2006; Braden et al., 2010, 2011; Lowry et al., 2010; but see Chisholm and Juraska, 2012). Here, using middle-aged surgically menopausal rats, we found that progesterone treatment transiently impaired working memory performance, and also resulted in a delay-induced impairment on the WRAM. Specifically, the present study replicates WRAM findings from Bimonte-Nelson et al. (2004b) whereby, after an initial learning block, progesterone-treated aged surgically menopausal rats committed more WMI errors across all trials and at the most demanding working memory load, as compared to vehicle-treated rats. Both progesterone-induced impairments in the current study were obviated by the addition of bicuculline. In addition, we found that progesterone, with or without bicuculline, had no impact on uterine weights or vaginal smears, supporting the lack of uterine stimulation expected with progesterone (Kirkland et al., 1992; Braden et al., 2010).

Although this is the third report of progesterone-induced cognitive impairments in middle-aged or aged Ovx rats (Bimonte-Nelson et al., 2004b; Braden et al., 2010), it is noted that overall the literature on progesterone effects on cognition in young Ovx rodents is mixed. Some studies show beneficial effects, mostly related to non-spatial memory (Frye et al., 2007; Frye and Walf, 2008; Harburger et al., 2008; Orr et al., 2009), while others show spatial memory impairments (Sun et al., 2010). When combined with 17β-estradiol, progesterone can abolish 17β-estradiol-induced memory improvements (Bimonte-Nelson et al., 2006; Harburger et al., 2007; Lowry et al., 2010; but see Gibbs, 2000; Markham et al., 2002) and can attenuate 17β-estradiol’s neurotrophic/neuroprotective effects *in vivo* (Bimonte-Nelson et al., 2004a; Rosario et al., 2006; Carroll et al., 2008), and in cell culture (Nilsen and Brinton, 2002a; Aguirre and Baudry, 2009). Further, administration of the progesterone metabolite, allopregnanolone, can impair cognition in young rats (Frye and Sturgis, 1995; Ladurelle et al., 2000; Johansson et al., 2002; Rabinowitz et al., 2014).

The goal of the current study was to assess the mechanism by which progesterone impairs memory, in the middle-aged surgically menopausal rat model, building on previous research that progesterone’s metabolites interact with the GABAergic system, acting as positive allosteric modulators (Paul and Purdy, 1992; Lan and Gee, 1994). Thus, we hypothesized that progesterone-mediated cognitive impairments may be initiated via pro-GABAergic actions, and that concomitant treatment with the GABA_A_ receptor antagonist, bicuculline, would block these impairments. In support of this hypothesis, we found that both the progesterone-induced impairment on working memory performance and delay retention, as evaluated by the WRAM, were reversed by the addition of bicuculline. Our findings are in line with a previous report of treatment with the GABA_A_ receptor antagonist, picrotoxin, attenuating the impairing effect of progesterone on an emotional learning task (Farr et al., 1995). The role of the GABAergic system in memory formation and retrieval has in part been delineated through administration of agonists and antagonists via systemic injections and local brain infusions (Izquierdo et al., 1993). An increase in GABA-mediated inhibition may impair memory on several levels. Briefly, findings show that GABAergic agonists inhibit the induction phase of LTP, and if administered post-training, can result in retrograde amnesia (Izquierdo et al., 1993).

The transient nature of the progesterone-impairing effect on learning is interesting, and may involve homeostatic plasticity changes via glutamatergic neurons. In addition to having pro-GABAergic actions, progesterone suppresses glutamate-induced excitation (Smith et al., 1987), protects against glutamate-induced toxicity (Nilsen and Brinton, 2002b), and decreases NMDA binding (Cyr et al., 2000). While to our knowledge the time course of the interplay between these opposing effects has not been extensively investigated for progesterone, this has been investigated for another ovarian hormone, 17β-estradiol. 17β-estradiol has been shown to alter the interplay between GABAergic and glutamatergic systems in a manner opposite to that of progesterone. 17β-estradiol suppresses GABAergic transmission while having pro-glutamatergic actions, which is a possible mechanism for dendritic spine formation and memory enhancement (Woolley et al., 1997; Bimonte and Denenberg, 1999; Rudick and Woolley, 2001; Segal and Murphy, 2001). After several days, 17β-estradiol-induced changes in GABAergic and glutamatergic systems ultimately result in a balance of excitation and inhibition, but possibly increase the long-term dynamic range of the system (Rudick and Woolley, 2001). Intuitively, the opposite pattern of effects by progesterone on the interplay between GABAergic and glutamatergic systems may decrease the dynamic range of the system. Indeed, progesterone has been shown to attenuate 17β-estradiol-mediated dendritic spine formation (Woolley and McEwen, 1993; Segal and Murphy, 2001; but see Gould et al., 1990). The behavioral outcome of reduced spine formation and a long-term “decreased dynamic range” of memory systems may be slowed learning, such as the transient effect of progesterone administration observed in the present study. This is in contrast to amnestic effects that persist through the entirety of maze testing, such as those observed with lesions to memory forming brain structures. Future research is necessary to delineate the time course of progesterone effects on the interplay between GABAergic and glutamatergic systems and how that might affect learning.

We have previously shown that a longer course (2 months) of progesterone or MPA treatment in aged Ovx rats decreased protein levels of CA1/2 hippocampal GAD (Braden et al., 2010). Wallis and Luttge (1980) found a similar effect of progesterone decreasing hippocampal GAD activity in young Ovx rats and others have found progesterone to alter mRNA levels of GAD (Weiland, 1992) and subunits of the GABA_A_ receptor (Weiland and Orchinik, 1995; Pazol et al., 2009) in the CA1. Similar changes in GAD and/or GABA_A_ receptor subunit expression may also be a mechanism of short-term progesterone-induced learning and memory impairments, and bicuculline reversal, observed herein. While this study implicates the GABAergic system in facilitating progesterone-induced cognitive impairments in the middle-aged Ovx rat, future investigations are warranted to further elucidate the neurochemical changes that accompany these cognitive impairments. This work is integral to the mechanistic understanding of progestogen-induced cognitive impairments in order to determine safe progestogen use, with respect to cognition, for combination birth control and HT p rescribed to women.

In conclusion, the current report indicates that: (1) short-term progesterone treatment transiently impairs working memory performance and results in a delay-induced impairment on the WRAM; and (2) these progesterone impairments are reversed by concomitant treatment with the GABA_A_ receptor antagonist, bicuculline. These findings support the hypothesis that progesterone-induced memory impairments in the middle-aged Ovx rat are in part mediated via pro-GABAergic actions.

## Conflict of Interest Statement

The authors declare that the research was conducted in the absence of any commercial or financial relationships that could be construed as a potential conflict of interest.
